# The associations of depression, anxiety, and insomnia at baseline with disability at a five-year follow-up point among outpatients with chronic low back pain: a prospective cohort study

**DOI:** 10.1186/s12891-023-06682-6

**Published:** 2023-07-11

**Authors:** Le-Yung Wang, Tsai-Sheng Fu, Mei-Chu Tsia, Ching-I Hung

**Affiliations:** 1 Department of Psychiatry, Chang Gung Memorial Hospital at Linkou and Chang Gung University College of Medicine, Taoyuan, Taiwan; 2 Department of Orthopedics, Chang Gung Memorial Hospital at Linkou and Chang Gung University College of Medicine, Taoyuan, Taiwan; 3 Department of Nursing, Chang Gung Memorial Hospital at Taoyuan and Chang Gung University of Science and Technology, Taoyuan, Taiwan

**Keywords:** Depressive disorder, Anxiety, Sleep, Chronic pain, Prognosis, Outcome

## Abstract

**Background:**

No previous study has investigated the associations of depression, anxiety, and insomnia at baseline with disability at a five-year follow-up point among outpatients with chronic low back pain (CLBP). The study aimed to simultaneously compare the associations of depression, anxiety, and sleep quality at baseline with disability at a 5-year follow-up point among patients with CLBP.

**Methods:**

Two-hundred and twenty-five subjects with CLBP were enrolled at baseline, and 111 subjects participated at the five-year follow-up point. At follow-up, the Oswestry Disability Index (ODI) and total months of disability (TMOD) over the past five years were used as the indices of disability. The depression (HADS-D) and anxiety (HADS-A) subscales of the Hospital Anxiety and Depression Scale and the Insomnia Severity Index (ISI) were used to assess depression, anxiety, and insomnia at baseline and follow-up. Multiple linear regression was employed to test the associations.

**Results:**

The scores of the HADS-D, HADS-A, and ISI were correlated with the ODI at the same time points (both at baseline and follow-up). A greater severity on the HADS-D, an older age, and associated leg symptoms at baseline were independently associated with a greater ODI at follow-up. A greater severity on the HADS-A and fewer educational years at baseline were independently associated with a longer TMOD. The associations of the HADS-D and HADS-A at baseline with disability at follow-up were greater than that of the ISI at baseline, based on the regression models.

**Conclusion:**

Greater severities of depression and anxiety at baseline were significantly associated with greater disability at the five-year follow-up point. The associations of depression and anxiety at baseline with disability at the long-term follow-up point might be greater than that of insomnia at baseline.

## Background

Back pain is a leading cause of disability [1] and the second most common reason for physician visits [2]. Predicting factors of chronic low back pain (CLBP) disability have been identified, such as body mass index (BMI), pain level, educational level, muscular strength and endurance, physical demands in the workplace, and social functioning [3–6]. Previous studies showed that depression, anxiety, and sleep problems are associated with disability [7–10] and work outcomes [11–13], and hinder recovery from disabling back pain [4, 14–17] among patients with CLBP.

The associations of depression, anxiety, and sleep quality at baseline with CLBP at long-term follow-up have not been clarified well. Most prospective studies followed-up the outcomes of CLBP with a short-term duration of 3 months to 2 years [8, 9, 11–13]. Although there are some studies with a follow-up duration longer than 2 years, these studies did not analyze depression, anxiety, and sleep problems simultaneously [5, 18]. Depression and anxiety are known to have long-term and fluctuating courses [19], and it is unclear whether they continue to impact disability at the 5-year follow-up point. Some cross-sectional studies have separately analyzed the relationships of depression, anxiety, and insomnia with CLBP [7, 20, 21], but they were not prospective and did not simultaneously investigate the impacts of depression, anxiety, and sleep quality among patients with CLBP.

Therefore, our study aimed to investigate the associations of depression, anxiety, and sleep quality at baseline with disability at a five-year follow-up point among patients with CLBP. The issue is important, because CLBP causes negative impacts on personal, social, and financial functioning [22], and understanding the important prognostic factors of disability is the first step by which to prevent it. We hypothesized that depression, anxiety, and sleep quality would be associated with disability at the five-year follow-up point among patients with CLBP; moreover, the impacts of depression and anxiety on disability might be greater than that of sleep quality.

## Methods

### Participants

The research was performed at the general orthopedics clinic in Chang Gung Memorial Hospital at Linkou, a medical center in Taiwan. At baseline (from August 2008 to November 2010), subjects were considered eligible if they were within 20–65 years of age and had suffered from LBP for at least 12 weeks, and had made a first visit to the orthopedics clinic. In this study, CLBP was defined as back pain with a duration of more than 12 weeks between the lower ribs and above the gluteal folds, with or without leg pain [23]. The exclusion criteria were patients who (1) had taken antipsychotics or antidepressants within the past one month, and (2) suffered from mental retardation, psychotic symptoms, or severe cognitive impairment with obvious difficulty in being interviewed. Physical examinations were performed and the findings of plain radiographs were diagnosed by a board-certified orthopedist after enrollment. Moreover, the subjects were interviewed by a board-certified psychiatrist who was blind to the data associated with CLBP. The board-certified psychiatrist used the Structured Clinical Interview for Diagnostic and Statistical Manual of Mental Disorders, fourth edition, text revision, Axis I Disorders to diagnose major depressive disorder (MDD) [24].

The program was an observational study. Treatment was not controlled, and the patients were treated as general orthopedics outpatients. Some patients discontinued treatment due to improvement or other reasons during the five-year period.

Five years later, the follow-up study was performed from August 2013 to July 2015. Written informed consent was obtained from all participants, based on the guidelines regulated in the Declaration of Helsinki, prior to study enrollment. The investigation was approved by the Institutional Review Board of the same hospital.

#### Assessment of depression, anxiety, sleep quality, and pain

The severities of depression and anxiety were evaluated using the Hospital Anxiety and Depression Scale (HADS) [25]. The HADS, which does not include any somatic symptoms and can assess depression and anxiety severity simultaneously, is composed of seven items for the depression (HADS-D) and anxiety (HADS-A) subscales [25, 26]. The total score of the HADS ranges from 0 to 21 for the two subscales.

The Insomnia Severity Index (ISI), a 7-item scale, has good reliability and validity to evaluate the severity of insomnia in the past two weeks [27, 28]. Each item of the ISI is rated on a 5-point scale and summed to generate a total score that ranges from 0 to 28. The visual analogue scale (VAS), with 0 representing “no pain” and 10 representing “pain as severe as I can imagine”, was used to evaluate the average pain intensity of LBP in the past 7 days. The HADS, ISI, and VAS were self-administered questionnaires completed by the participants. Higher scores on the HADS-D, HADS-A, and ISI indicate a greater severity of symptoms.

### Assessment of disability

Disability associated with CLBP was measured using the Oswestry Disability Index (ODI), which was considered as the major outcome. Moreover, self-reported total months of disability (TMOD) due to CLBP over the past 5 years was used as the secondary outcome.

The Oswestry Disability Questionnaire, which includes 10 items, evaluates back or leg pain-related disability in daily life [29, 30]. The score of the questionnaire ranges from 0 to 50, and is usually multiplied by 2 to obtain the ODI. The ODI was used as the primary outcome in this study because it is of sufficient reliability and validity, and is the most commonly-used functional outcome tool for LBP [30].

Patients were requested to report TMOD in employment and/or domestic work due to LBP over the past 5 years. The self-reported TMOD was used because the ODI evaluates the degree of disability in recent daily activities at the follow-up point and the TMOD represents the total duration of disability over the past five years.

### Statistical methods

All statistical analyses were performed using SPSS (International Business Machines Corporation, United States) version 20.0 for Windows. The independent t-test, paired t-test, Chi-square test, Pearson’s correlation, and McNemar’s test were used appropriately. Multiple linear regression with the Forward method was employed to determine the associations of severities of depression, anxiety, and insomnia at baseline with the two indices of disability at the follow-up point. The first and second models tested the associations of depression, anxiety, and insomnia with the ODI and TMOD, respectively. Therefore, the dependent variables in the first and second models were the ODI and TMOD, respectively. In the first model, the independent variables included five demographic variables (age, gender, educational years, marital status, and employment status) at baseline and 10 other variables at baseline, including the HADS-D and HADS-A scores, ISI score, ODI, pain intensity (VAS), with life-time MDD history or not, with past surgical history or not, with abnormal radiographic findings or not, with obesity (BMI ≥ 25) [31] or not, and with associated leg symptoms or not. In the second model, the independent variables were the same as in the first model. In all statistical analyses, a two-tailed test with a *P* value < 0.05 was considered to indicate statistical significance.

## Results

### Subjects

At baseline, 225 patients were enrolled (122 males and 103 females) in the study. During the 5-year period, some patients stopped treatment or dropped out. At the 5-year follow-up point, 111 (49.3%) patients, including 46 women and 65 men, agreed to participate and 114 (50.7%) patients did not attend follow-up for the following reasons: 35 (15.6%) could not be contacted by mail or phone; 79 (35.1%) refused to enter the follow-up program.

There were no significant differences in demographic variables at baseline, except for marital status, between the patients with and without follow-up (Table 1). There were also no significant differences in the severities of depression, anxiety, sleep quality, pain intensity, and disability (ODI score), nor the percentages of other clinical variables, at baseline between the two subgroups. At the follow-up point, the TMOD was 9.1 ± 17.5 months among those who participated at follow-up.

**Table 1:** Demographic variables and psychometric scores at baseline and follow-up^a^

	Baseline			Follow-up
	Total (n = 225)	Without follow-up (n = 114)	With follow-up (n = 111)	(n = 111)
Age (years)	40.7 ± 11.4	39.7 ± 11.1	41.7 ± 11.7	46.7 ± 11.7
Education (years)	11.4 ± 3.4	11.1 ± 3.4	11.6 ± 3.4	11.6 ± 3.4
Female (%)	45.8	50.0	41.4	41.4
Married (%)	69.3	63.2*	75.7	79.3
Employment (%)	67.6	68.4	66.7	69.4
Radiographic findings (%)	66.2	61.4	71.2	73.0
Associated leg symptoms (%)	42.7	42.1	43.2	55.0*
Major depressive disorder (%)	21.8	22.8	20.7	29.7**
ISI	9.7 ± 6.7	10.5 ± 6.5	8.9 ± 6.9	7.9 ± 6.1
Past surgical history (%)	5.3	3.5	7.2	25.2**
Height (cm)	164.2 ± 8.4	163.8 ± 8.1	164.6 ± 8.7	164.4 ± 8.9
Weight (kg)	65.9 ± 12.6	65.0 ± 12.4	66.7 ± 12.9	68.4 ± 13.0**
BMI	24.3 ± 3.5	24.1 ± 3.5	24.5 ± 3.6	25.2 ± 3.6**
Obesity (BMI ≥ 25) (%)^b^	42.7	43.0	42.3	53.2**
Pain intensity ^c^	5.7 ± 2.7	5.8 ± 2.6	5.7 ± 2.7	2.6 ± 2.7**
ODI	31.4 ± 15.3	32.0 ± 16.2	30.8 ± 14.4	18.3 ± 14.4**
HADS-D	5.8 ± 4.1	5.9 ± 4.2	5.7 ± 4.1	4.8 ± 3.7*
HADS-A	7.3 ± 4.4	7.8 ± 4.7	6.8 ± 4.1	5.2 ± 3.7**

^a^ Statistical differences in continuous and categorical variables between subjects with and without follow-up were examined by the independent t-test and Chi-square test, respectively. Statistical differences in continuous and categorical variables for subjects with follow-up at the two time points were investigated by the paired t-test and *McNemar’s* test, respectively

^b^ Obesity was defined as BMI ≥ 25

^c^ Pain intensity was evaluated using a 0–10 visual analogue scale

**ISI** = Insomnia Severity Index, **ODI** = Oswestry Disability Index, **HADS-D** = Depression subscale of the Hospital Anxiety and Depression Scale (HADS), **HADS-A** = Anxiety subscale of the HADS, **TMOD** = self-reported total months of disability over the past 5 years

*p < 0.05, **p < 0.01

Among the 111 subjects with follow-up, abnormal radiographic findings included spondylosis without instability (47.7% and 37.8% at baseline and follow-up, respectively), spondylolisthesis (13.5% and 17.1%), spondylolysis (13.5% and 10.8%), scoliosis (5.4% and 8.1%), and other findings (12.6% and 28.8%). In the follow-up subgroup, 28 (25.2%) subjects accepted surgical operations for CLBP. Over the five years, 5 (4.5%) subjects were treated in psychiatric clinics. Over the past one year, 25 and 42 (22.5% and 37.8%) subjects accepted treatment in rehabilitation and traditional Chinese medicine clinics, respectively, and 32 (28.2%) subjects took analgesics for CLBP.

### Differences in clinical variables at baseline and follow-up among subjects who participated at follow-up

Table 1 shows a significantly decreased pain intensity (p < 0.001), ODI score (p < 0.001), HADS-D score (p = 0.03), and HADS-A score (p < 0.001) at the follow-up point as compared with baseline. There was an increased mean value of BMI (p < 0.001), and increased percentages of obesity (p = 0.003), life-time history of MDD (p < 0.01), associated leg symptoms (p = 0.02), and past history of surgical operation (p < 0.001) at follow-up.

### Differences in disability, depression, and anxiety at the follow-up point between subjects with and without clinical variables at baseline

Table 2 shows that subjects with MDD at baseline had a significantly higher ODI (p = 0.03) and significantly higher severities of depression (p < 0.01), anxiety (p = 0.03), and insomnia (p < 0.01) at the follow-up point. Subjects with a history of surgical operation and associated leg symptoms at baseline had a significantly higher depressive severity (p = 0.02) and ODI (p = 0.01) at the follow-up point, respectively.

**Table 2:** Mean values (± standard deviation) of the severities of disability, depression, and anxiety, and duration of disability in the subgroups^a,b^

		ODI_(5Y)_	TMOD_(5Y)_	HADS-D_(5Y)_	HADS-A_(5Y)_	Pain intensity ^c^	ISI_(5Y)_
Female	Yes (n = 46)	19.8 ± 13.9	10.2 ± 18.6	4.7 ± 4.1	4.6 ± 3.7	2.7 ± 2.8	7.6 ± 5.5
	No (n = 65)	17.2 ± 14.7	8.4 ± 16.7	4.9 ± 3.4	5.7 ± 3.7	2.6 ± 2.7	8.2 ± 6.5
Radiographic findings_(B)_	Yes (n = 79)	19.3 ± 15.1	9.5 ± 17.9	4.8 ± 3.5	5.2 ± 3.5	2.6 ± 2.8	7.9 ± 6.2
	No (n = 32)	15.8 ± 12.0	8.2 ± 16.6	4.9 ± 4.4	5.3 ± 4.2	2.6 ± 2.7	7.9 ± 5.7
Associated leg symptoms_(B)_	Yes (n = 48)	22.4 ± 17.1*	11.9 ± 19.5	5.1 ± 3.6	5.6 ± 3.7	2.8 ± 2.8	8.9 ± 6.6
	No (n = 63)	15.2 ± 11.0	7.0 ± 15.6	4.6 ± 3.8	5.0 ± 3.8	2.5 ± 2.7	7.2 ± 5.6
Major depressive disorder_(B)_	Yes (n = 23)	25.4 ± 17.5*	14.2 ± 22.9	6.8 ± 4.7**	6.7 ± 3.6*	3.7 ± 3.1	11.3 ± 5.4**
	No (n = 88)	16.4 ± 12.9	7.8 ± 15.7	4.3 ± 3.3	4.8 ± 3.7	2.3 ± 2.6	7.1 ± 5.9
Past surgical history_(B)_	Yes (n = 8)	30.0 ± 21.9	23.8 ± 26.1	7.8 ± 4.8*	6.5 ± 4.9	3.8 ± 3.1	7.9 ± 7.5
	No (n = 103)	17.4 ± 13.3	8.0 ± 16.3	4.6 ± 3.6	5.1 ± 3.6	2.5 ± 2.7	7.9 ± 6.0
Obesity (BMI ≥ 25)_(B)_	Yes (n = 47)	19.8 ± 15.5	19.7 ± 25.7	4.8 ± 3.5	4.8 ± 3.6	2.5 ± 2.7	7.9 ± 6.3
	No (n = 64)	17.2 ± 13.5	14.5 ± 23.2	4.9 ± 3.9	5.5 ± 3.8	2.7 ± 2.8	8.0 ± 5.9

^a^ “B” and “5Y” represent data collected at baseline and at the 5-year follow-up point, respectively

^b^ Differences in scores between subgroups were measured by the independent sample t-test

^c^ Pain intensity was evaluated using a 0–10 visual analogue scale

**ODI** = Oswestry Disability Index, **HADS-D** = Depression subscale of the Hospital Anxiety and Depression Scale (HADS), **HADS-A** = Anxiety subscale of the HADS, **TMOD** = self-reported total months of disability over the past 5 years, **ISI** = Insomnia Severity Index

*p < 0.05, **p < 0.01

### Correlations of depression, anxiety, and insomnia with the ODI at the same time points

At baseline, the severities of depression (r = 0.46, p < 0.001), anxiety (r = 0.30, p < 0.01), and sleep quality (r = 0.26, p < 0.01) were correlated with the ODI at the same time point among those who participated at follow-up (n = 111). At follow-up, the severities of depression (r = 0.41, p < 0.001), anxiety (r = 0.32, p = 0.001), and sleep quality (r = 0.54, p < 0.001) were also correlated with the ODI at the same time point.

### Correlations of continuous variables at baseline with disability, depression, and anxiety at the follow-up point

The correlations of all clinical continuous variables, except for the ISI at baseline, with the ODI at follow-up were significant (P < 0.05) and slightly correlated (r = 0.2–0.4) (Table 3). Educational years (p = 0.001) and the severity of depression (p = 0.04) were slightly correlated with the TMOD at follow-up. The severities of depression, anxiety, pain intensity, and sleep quality at baseline were significantly correlated with the same indices at follow-up.

**Table 3:** Correlations of clinical variables at baseline with disability, depression, and anxiety at follow-up^a,b^

	ODI_(5Y)_	TMOD_(5Y)_	HADS-D_(5Y)_	HADS-A_(5Y)_	Pain intensity_(5Y)_^c^	ISI_(5Y)_
Age_(B)_	0.31**	0.13	-0.11	-0.23*	0.08	-0.05
Educational years_(B)_	-0.28**	-0.32**	-0.05	0.06	-0.08	-0.04
BMI_(B)_	0.23*	0.14	0.12	-0.01	0.06	0.12
Pain intensity_(B)_	0.22*	0.09	0.31**	0.27**	0.37**	0.26**
ODI_(B)_	0.28**	0.18	0.25**	0.09	0.17	0.19*
HADS-D_(B)_	0.33**	0.20*	0.44**	0.33**	0.15	0.35**
HADS-A_(B)_	0.20*	0.17	0.44**	0.52**	0.17	0.35**
ISI_(B)_	0.11	0.16	0.42**	0.38**	0.25**	0.46**

^a^ “B” and “5Y” represent data collected at baseline and at the 5-year follow-up point, respectively

^b^ These correlation coefficients were measured using Pearson’s correlation

^c^ Pain intensity was evaluated using a 0–10 visual analogue scale

**ODI** = Oswestry Disability Index, **HADS-D** = Depression subscale of the Hospital Anxiety and Depression Scale (HADS), **HADS-A** = Anxiety subscale of the HADS, **TMOD** = self-reported total months of disability over the past 5 years, **ISI** = Insomnia Severity Index

*p < 0.05, **p < 0.01

### Independent factors at baseline associated with disability at follow-up

Table 4 shows that subjects with a greater severity of depression, an older age, and associated leg symptoms at baseline were independently associated with a higher ODI at the follow-up point. The first regression model demonstrated that increases of one point on the HADS-D and one year of age at baseline were associated with increases of 1.15 and 0.34 points on the ODI at follow-up, respectively. Moreover, the HADS-D, age, and with associated leg symptoms could explain 11%, 10%, and 5% of the variance in the ODI at follow-up, respectively. The severity of depression had the highest R square change (R square = 0.11). The second regression model showed that fewer educational years and a higher anxiety severity were independently associated with a higher TMOD. Increases of one year of education and one point on the HADS-A at baseline were associated with a decrease of 1.94 and an increase of 1.08 months of TMOD at follow-up, respectively. Educational years and the HADS-A score could explain 10% and 6% of the variance in the TMOD at follow-up, respectively.

**Table 4:** Independent factors related to disability among outpatients with chronic low back pain^a^

	Independent variables	B	95% CI	t	R^2^ change	p value
ODI_(5Y)_	HADS-D_(B)_	1.15	0.57 to 1.73	3.9	0.11	< 0.001
	Age_(B)_	0.34	0.14 to 0.55	3.3	0.10	0.001
	Associated leg symptoms_(B)_	6.18	1.35 to 11.0	2.5	0.05	0.013
TMOD_(5Y)_	Educational years_(B)_	-1.94	-2.86 to -1.02	-4.2	0.10	< 0.001
	HADS-A_(B)_	1.08	0.31 to 1.85	2.8	0.06	0.006

^a^ “B” and “5Y” represent data collected at baseline and at the 5-year follow-up point, respectively

**ODI** = Oswestry Disability Index, **HADS-D** = Depression subscale of the Hospital Anxiety and Depression Scale (HADS), **HADS-A** = Anxiety subscale of the HADS, **TMOD** = self-reported total months of disability over the past 5 years

## Discussion

The first regression model demonstrated that a greater severity of depression at baseline was related to a higher ODI at the 5-year follow-up point. Table 3 shows that the correlation of the HADS-D at baseline with the ODI at follow-up had the highest correlation co-efficiency as compared with other variables at baseline with the ODI at follow-up. Previous studies also showed that depression at baseline was associated with a poorer functional outcome and less possibility of recovering from CLBP [5, 8, 9, 18]. Our results further demonstrated that depression had chronic and long-term negative impacts on the prognosis of CLBP at the 5-year follow-up point.

Depression may be responsible for the development and maintenance of self-reported pain and disability [32, 33], which removes the ability of patients to cope with physical problems. Besides, symptoms of depression such as negative perceptions, especially fear and catastrophizing, anhedonia, fatigue, psychomotor retardation, and social-behavioral withdrawal, are significantly mediated by the relationship between pain and disability, and affect the capability of returning to daily life or work [7, 34, 35]. Another theory suggests that some genetic factors are associated with the relationship between lifetime LBP and depression [36, 37].

The second regression model showed that a greater severity of anxiety at baseline was also associated with a longer TMOD at follow-up. Anxiety increases the risk of acute LBP developing into CLBP [38], and is meanwhile a barrier for treatment adherence in chronic pain conditions [4]. A higher level of anxiety or pain catastrophizing is associated with the fear avoidance model and induces pain-related functional disability [39–41]. Our results demonstrated that anxiety at baseline was also an important factor associated with long-term negative outcomes among patients with CLBP.

Several points were worthy of note: (1) The severities of depression, anxiety, and insomnia were correlated with the ODI at the same time points. This was compatible with previous studies [15, 17]. However, the regression model showed that insomnia at baseline was not a significant factor associated with the two indices of disability at follow-up. This might demonstrate that the severities of depression and anxiety at baseline had a higher power to predict disability at follow-up as compared with insomnia at baseline. (2) The second regression model showed that greater educational years was associated with a lower TMOD (Table 4). CLBP patients with a lower educational level have a higher chance of obtaining a job with a greater physical workload [42, 43], which may interfere with treatment adherence [44], the severity of CLBP symptoms, and the possibility of returning to work [45]. Moreover, CLBP patients with a lower educational level might find health information to be less accessible [46]. (3) The regression model demonstrated that associated leg symptoms at baseline was related to a higher ODI at follow-up. Previous study showed that CLBP patients with associated leg symptoms had a poorer prognosis, higher disability, and a poorer quality of life, and used more health resources as compared with those without associated leg symptoms [47].

There were several limitations of this study. (1) All subjects were enrolled from one medical center. Our study had a smaller sample size as compared with one previous long-term follow-up study of patients with CLBP [5]. Although there were no significant differences in the severities of the ODI, pain intensity, HADS-D, HADS-A, and ISI at baseline between subjects with and without follow-up, unknown bias might exist and cause confounding effects. Therefore, expansion of the results to the general population should be performed cautiously. (2) The TMOD was a self-reported measure and might have been affected by recall bias. In fact, one previous study reported recall bias to be an important confounding factor [48], especially under a long recall period. Therefore, interpretation of our results should be performed carefully. (3) Subjects’ treatments were not controlled over the five years. Time-series data on interventions for CLBP were not available. There was a possibility that different treatments might have interfered with the long-term outcomes.

## Conclusion

Greater severities of depression and anxiety at baseline were significantly associated with a higher ODI and a longer TMOD at the five-year follow-up point, respectively. Even controlling for the ODI at baseline, the severity of depression at baseline was still associated with the ODI at follow-up. The associations of depression and anxiety at baseline with disability at the long-term follow-up point might be greater than that of insomnia at baseline.

## Data Availability

The datasets in the current study are available on reasonable request from the corresponding author.

## Abbreviations

CLBP: Chronic low back pain
BMI: Body mass index
LBP: Low back pain
MDD: Major depressive disorder
HADS-D: Depression subscale of the Hospital Anxiety and Depression Scale
HADS-A: Anxiety subscale of the HADS
ISI: Insomnia Severity Index
VAS: Visual analogue scale
ODI: Oswestry Disability Index
TMOD: Total months of disability
